# Poster Session II – Poster of Distinction II A320 3D IMAGING UNCOVERS STRUCTURAL ALTERATIONS IN THE MUSCULARIS MACROPHAGES AND ICC NETWORK ASSOCIATED WITH MICROBIOTA-MEDIATED GUT DYSFUNCTION

**DOI:** 10.1093/jcag/gwaf042.320

**Published:** 2026-02-13

**Authors:** E Giordano, C Shimbori, J Lu, G De Palma, X Bai, S Collins, P Bercik

**Affiliations:** McMaster University, Hamilton, ON, Canada; McMaster University, Hamilton, ON, Canada; McMaster University, Hamilton, ON, Canada; McMaster University, Hamilton, ON, Canada; McMaster University, Hamilton, ON, Canada; McMaster University, Hamilton, ON, Canada; McMaster University, Hamilton, ON, Canada

## Abstract

**Background:**

*Clostridioides difficile* infection (CDI) is common and often triggered by gut microbiota disruption following antibiotic exposure. Up to 25% of patients develop persistent gut dysfunction after CDI eradication, including constipation, the mechanisms of which remain unclear. Healthy colonic peristalsis results from complex interplay between interstitial cells of Cajal (ICC), muscle and enteric nervous systems, with input from immune cells, including muscularis macrophages (MMØ). We found that microbiota from patients who developed severe constipation following CDI induces slow colonic transit in germ-free mice, with MMØ playing an important role. Here we use confocal imaging to investigate whether microbiota from pCDI patients alter the ICC network and MMØ morphology.

**Aims:**

To determine whether pCDI microbiota induce structural alterations in the MMØ and ICC network.

**Methods:**

Germ-free NIH Swiss mice (n = 27) were colonized with fecal microbiota from 3 pCDI patients with severe constipation or 3 healthy controls. Three weeks later, colonic transit was assessed via bead expulsion. Colonic tissues were stained for c-kit^+^ ICC and CD68^+^ macrophages and imaged by 3D confocal microscopy (Nikon A1R). Image quantification and morphological analyses were performed using FIJI (ImageJ) and RStudio pipelines, with statistical analyses in GraphPad Prism (v10.4.1). Data are expressed as mean ±SEM and analyzed by Mann-Whitney or Kolmogorov-Smirnov tests.

**Results:**

Mice with pCDI microbiota exhibited slower colonic transit than those with control microbiota (398 ±51.5 vs. 150 ±30.2 seconds; *p*<0.001*)*. Confocal imaging revealed disrupted ICC networks in the proximal colon, with fewer branches (15021 ±1358 vs. 21720 ±2573; *p < *0.05*)* and junctions (6509 ± 657.4 vs. 9768 ±1243; *p*<0.05*)*. MMØ displayed morphological alterations, including increased cell area (5312 ±213 vs. 4411 ±241; *p*<0.05), max branch length (16.7 ±0.293 vs. 15.1 ±0.428; *p < 0.05*), bounding circle diameter (130 ±2.24 vs. 114 ±2.63; *p < *0.001), perimeter (309 ±5.50 vs. 274 ±6.29;*p < *0.001) and reduced circularity (0.66 ±0.01 vs. 0.69 ± 0.01; *p*<0.01). Spatial co-localization showed broader ICC-MMØ overlap in mice with pCDI microbiota compared to controls (range =0.04 vs. 0.02; *p*<0.05).

**Conclusions:**

Microbiota from pCDI patients induce a slow-transit phenotype in gnotobiotic mice, associated with structural remodelling of gut pacemaker system and muscularis macrophages. These findings support a role for microbiota-driven immune mechanism underlying post-infectious gut dysfunction.

**Funding Agencies:**

CIHR

